# Enhancing TB Case Detection: Experience in Offering Upfront Xpert MTB/RIF Testing to Pediatric Presumptive TB and DR TB Cases for Early Rapid Diagnosis of Drug Sensitive and Drug Resistant TB

**DOI:** 10.1371/journal.pone.0105346

**Published:** 2014-08-20

**Authors:** Neeraj Raizada, Kuldeep Singh Sachdeva, Sreenivas Achuthan Nair, Shubhangi Kulsange, Radhey Shayam Gupta, Rahul Thakur, Malik Parmar, Christen Gray, Ranjani Ramachandran, Bhavin Vadera, Shobha Ekka, Shikha Dhawan, Ameet Babre, Mayank Ghedia, Umesh Alavadi, Puneet Dewan, Mini Khetrapal, Ashwini Khanna, Catharina Boehme, Chinnambedu Nainarappan Paramsivan

**Affiliations:** 1 Foundation for Innovative New Diagnostics, New Delhi, India; 2 Central TB Division, Government of India, New Delhi, India; 3 World Health Organization, Country Office for India, New Delhi, India; 4 Foundation for Innovative New Diagnostics, Geneva, Switzerland; 5 District Tuberculosis Center, Mumbai, India; 6 District Tuberculosis Center, New Delhi, India; Hopital Raymond Poincare - Universite Versailles St. Quentin, France

## Abstract

**Background:**

Diagnosis of pulmonary tuberculosis (PTB) in children is challenging due to difficulties in obtaining good quality sputum specimens as well as the paucibacillary nature of disease. Globally a large proportion of pediatric tuberculosis (TB) cases are diagnosed based only on clinical findings. Xpert MTB/RIF, a highly sensitive and specific rapid tool, offers a promising solution in addressing these challenges. This study presents the results from pediatric groups taking part in a large demonstration study wherein Xpert MTB/RIF testing replaced smear microscopy for all presumptive PTB cases in public health facilities across India.

**Methods:**

The study covered a population of 8.8 million across 18 programmatic sub-district level tuberculosis units (TU), with one Xpert MTB/RIF platform established at each study TU. Pediatric presumptive PTB cases (both TB and Drug Resistant TB (DR-TB)) accessing any public health facilities in study area were prospectively enrolled and tested on Xpert MTB/RIF following a standardized diagnostic algorithm.

**Results:**

4,600 pediatric presumptive pulmonary TB cases were enrolled. 590 (12.8%, CI 11.8–13.8) pediatric PTB were diagnosed. Overall 10.4% (CI 9.5–11.2) of presumptive PTB cases had positive results by Xpert MTB/RIF, compared with 4.8% (CI 4.2–5.4) who had smear-positive results. Upfront Xpert MTB/RIF testing of presumptive PTB and presumptive DR-TB cases resulted in diagnosis of 79 and 12 rifampicin resistance cases, respectively. Positive predictive value (PPV) for rifampicin resistance detection was high (98%, CI 90.1–99.9), with no statistically significant variation with respect to past history of treatment.

**Conclusion:**

Upfront access to Xpert MTB/RIF testing in pediatric presumptive PTB cases was associated with a two-fold increase in bacteriologically-confirmed PTB, and increased detection of rifampicin-resistant TB cases under routine operational conditions across India. These results suggest that routine Xpert MTB/RIF testing is a promising solution to present-day challenges in the diagnosis of PTB in pediatric patients.

## Background

Prevalence of childhood TB is a reflection of recent transmission and serves as an important epidemiological indicator [1]. Childhood TB accounts for 6-10% of all TB cases worldwide, thereby continuing to be an important cause of morbidity and mortality in this group [2]–[3]. India has one of the highest burdens of TB in the world [4]. The number of pediatric TB cases registered under India's Revised National Tuberculosis Control Programme (RNTCP) has shown a constant level trend in the past five years. In 2012, 81,482 pediatric cases were notified accounting for 7% of all notified TB cases [5]. While the exact burden of childhood TB globally is not known due to diagnostic difficulties, it is estimated that childhood TB constitutes about 10–20% of all TB, in high burden countries [6]–[7].

Accurate diagnosis of pediatric TB remains an impediment in the management of pediatric TB. The diagnosis is complicated because children are unable to expectorate sputum and TB can mimic many other common childhood diseases including pneumonia, generalized bacterial and viral infections, and malnutrition and respiratory opportunistic infections associated with HIV [8]. Bacteriological confirmation of infection in sputum, gastric, tracheal, or bronchial aspirates and in other body fluids in infants and children is challenging because of difficulties obtaining samples and due to the paucibacillary nature of disease [9]. Even under optimal circumstances, the sensitivity of smear microscopy for the diagnosis of childhood TB remains low [8]–[10]. In the absence of bacteriological confirmation, the diagnosis of childhood TB in countries where TB is not endemic is based on a triad of close contact with an infectious patient, a positive tuberculin skin test (TST) result, and presence of suggestive abnormalities on a chest radiograph. These criteria, however, have limited application in countries where TB is endemic and many individuals acquire infection and become TST positive during childhood and adolescence. Although liquid culture is considered to be the most sensitive test for diagnosis of active TB and drug susceptibility, access to liquid culture for TB diagnosis is extremely limited in resource-poor settings [9]. These challenges in establishing accurate diagnosis of TB in children add to the potential for both under and over-diagnosis based on clinical judgment and non-specific tools like X-ray [11].

Xpert MTB/RIF, a highly sensitive and specific tool with a quick turn-around time, offers a promising solution in addressing these challenges in the diagnosis of pediatric PTB. We report here our experiences of offering upfront Xpert MTB/RIF to pediatric presumptive PTB cases for early diagnosis of TB and drug-resistant TB (DR-TB) in the programmatic settings at peripheral level amongst this vulnerable population in India. The objectives of the study were to assess the effect of Xpert MTB/RIF substitution for smear microscopy in public clinics on the diagnosis of pediatric PTB, bacteriologically-confirmed PTB, and rifampicin-resistant TB and to assess the PPV of detection of rifampicin resistance by Xpert MTB/RIF in this pediatric patient population.

## Methods

### Setting

With a population of about 1.2 billion, India has 662 district TB programme units and 2698 sub-district TB programme units referred to as Tuberculosis units (TUs), each TU covering a population of approximately 0.5 million. The present study was conducted in 18 selected TUs. Study TUs were selected by a national committee to be indicative of a broad diversity of settings relevant for TB control practice including geographic area, urban/rural composition, and TB burden. Furthermore, they were restricted to those with free treatment available for patients diagnosed with rifampicin-resistant TB. Each of the 18 study TUs have 4–6 designated microscopy centres (DMCs), each covering a population of approximately 0.1 million where sputum smear microscopy is available for PTB diagnosis. Three to five primary health centres are linked to each microscopy centre, referring presumptive PTB cases to the respective microscopy centre.

**Figure 1:**
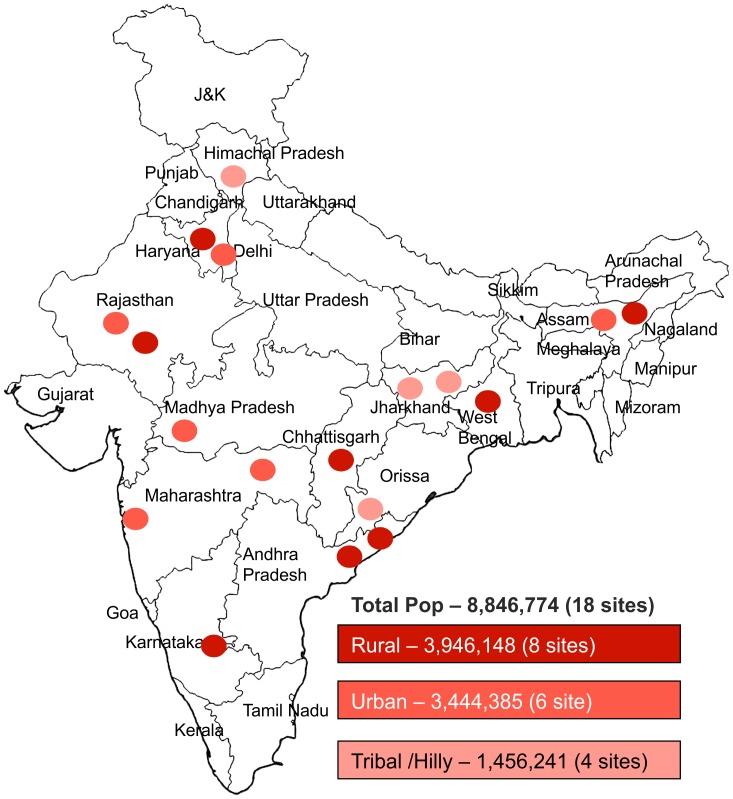
Geographical location of study treatment units and the demographic classification assigned to each project treatment unit site.

Of these 18 study TUs, 8 sites were rural, 6 sites were urban and the remaining 4 sites were tribal covering a population of 3.9 million, 3.4 million, and 1.5 million, respectively. (Figure 1) Altogether, these 18 sites account for 8.8 million people having access to TB diagnostic services through 99 DMCs and their corresponding linked health facilities. An Xpert MTB/RIF platform was established at the existing laboratory in the largest community health center or hospital in each TU. The catchment included all public health facilities in the geographic area by means of specimen transportation linkages which were established for study.

### Definitions

Presumptive pediatric PTB cases were defined as per the Indian Revised National TB Control Programme (RNTCP) guidelines [12]–[13]. This includes children presenting with fever and/or cough for ≥2 weeks, with or without weight loss or no weight gain, or showing neurological symptoms like irritability, refusal to eat, headache, and vomiting.

Presumptive pediatric DR-TB cases were defined as previously-diagnosed pediatric PTB cases based on smear-microscopy referred for drug susceptibility testing (DST) because of an elevated risk of drug-resistant TB. National guidelines used in the study define high-risk TB cases as those with previous history of anti-TB treatment, on treatment with positive sputum smear result at any follow up smear examination, diagnosed TB cases with HIV-co-infection, and pulmonary TB cases who are contacts of a known MDR-TB case [14].

Bacteriologically-confirmed PTB cases were defined as having a sputum specimen positive by smear microscopy, culture or Xpert MTB/RIF, or other WHO-approved rapid diagnostic test [15].

Clinically diagnosed PTB cases are defined as cases that do not fulfill the criteria for bacteriological confirmation but are diagnosed with active TB by a treating physician using a standardized programmatic diagnostic algorithm which incorporates chest X-ray, antibiotic trial, repeat smear microscopy and clinical evaluation of symptoms and are initiated on TB treatment, as evidenced by registration in a RNTCP treatment register [15].

PTB cases included any bacteriologically-confirmed or clinically diagnosed case of TB involving the lung parenchyma or the tracheo-bronchial tree. PTB cases were defined as those patients with at least one of two smears positive for acid fast bacilli (AFB) using direct microscopy or a patient with symptoms suggestive of TB with two smear examination negative for AFB, with evidence of pulmonary TB by another microbiological method (culture positive or by other approved molecular methods) or chest X-ray [12].

### Design

**Figure 2:**
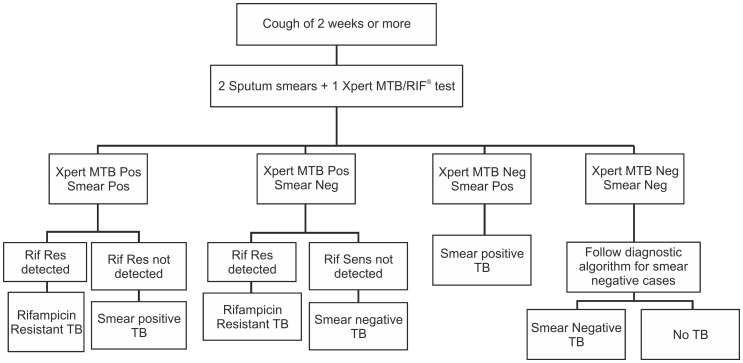
Diagnostics Algorithm used under the study.

Children (age 0-14 yrs) presenting with signs and symptoms suggestive of PTB to any of the public health facility in the study areas were prospectively enrolled in the project. Two sputum specimens were collected at microscopy centres and transported to the respective Xpert MTB/RIF lab. In children of less than 6 years where two sputum specimens could not be collected, induced sputum (IS) or nasopharyngeal aspirated was collected in line with the existing practices and guidelines of the RNTCP. The procedure used involved administration of an inhaled bronchodilator followed by nebulized hypertonic (3–5%) saline then nasopharyngeal aspiration or expectoration of mucus from the lower respiratory tract [16]. As per the project diagnostic algorithm (figure 2), Both specimens (spot and morning) were subjected to Ziehl Neelsen (ZN) smear microscopy and one of the two samples was tested on Xpert MTB/RIF, the first available specimen being tested on Xpert MTB/RIF.

The current study was carried out in uncontrolled programmatic field settings with the key project intervention being upfront testing on Xpert MTB/RIF. As the study covered all health facilities in the selected geographic area, no patient related clinical data, including details of X-Ray, histo-pathological findings, etc was collected. To minimize the delay in reporting Xpert MTB/RIF test results were relayed by Short Messaging Service (SMS) to all health providers and referring facilities. Under the study, universal access to Xpert MTB/RIF testing was provided to patients coming to health facilities irrespective of whether their place of residence fell within the study TU or not. As per the existing RNTCP structure and guidelines, a given TU maintains records and undertakes active management and follow-up of diagnosed TB cases that are initiated on treatment at facilities within the same TU area. Under the current study, all consecutive presumptive cases were provided access to project intervention; however, with a small study team, collecting and compiling information on all treatment initiation of diagnosed TB cases that resided outside the study TU was not within the scope of the current study. However, the respective programme officials of the area in which the diagnosed TB cases resided were informed on the details of such cases, as per the RNTCP guidelines and by SMS.

Treatment of PTB and rifampicin-resistant TB was initiated based on Xpert MTB/RIF results in line with the project diagnostic algorithm as approved by RNTCP as part of the programme services. Any presumptive PTB case receiving a negative result on Xpert MTB/RIF and a positive result on smear microscopy was managed based on results of smear microscopy. Patients diagnosed as rifampicin-resistant on Xpert MTB/RIF had an additional specimen collected and sent to the regional RNTCP DST laboratories for solid/liquid media DST and line probe assay (LPA) for confirmation. Treatment of these cases was based on initial Xpert MTB/RIF rifampicin resistance results. Any case with rifampicin susceptible results on solid/liquid media DST was subsequently switched to appropriate regimen based on the decision of treating physicians.

### Data management

Data for all presumptive PTB and DR-TB cases were collected using standardized case report forms (CRFs) by the RNTCP staff working at microscopy centres and TUs. Data from CRFs were entered using a secure, web-based MIS (Management Information System) by the site staff. Quality of data was ensured by regular cross-validation against programme records by project supervisors. Data was extracted and analyzed using EpiData Analysis (Version 2.1) and Microsoft Excel 2007. All confidence intervals were calculated based on the binomial distribution with a 95% probability interval. For analysis, pediatric patients were categorized into 3 groups: 0–4 years, 5–9 years, and 10–14 years of age.

Annualized rates of bacteriologically-confirmed PTB case detection in the study were compared with reported program data from 2011 in terms of number of cases per 100,000 persons per year to account for the variable population coverage and duration of data collection at each site. Student's paired T-test was applied to determine whether differences between the two data sets were statistically significant.

### Ethical issues

The study protocol was approved by the Institution Ethics Committee of the National Tuberculosis Institute, Bangalore, India. Structured informed consent forms were used for obtaining written informed consent from parents, caretakers, or guardians on behalf of the minors/children enrolled in the study as approved in the ethical approval. Before consenting, patients were informed about the study in vernacular language by trained staff. For illiterate patients, after explaining verbally, the consent was taken in presence of literate witness. Approval for the research was granted by the Central TB Division, Ministry of Health and Family Welfare, Government of India.

### Funding source

The study was funded by United States Agency for International Development (USAID) and facilitated by the World Health Organization (WHO). FIND was responsible for study design, implementation, training, study coordination and monitoring, data analysis, and writing of the report in close coordination with WHO and Central TB Division. The funder had no participation in the design, implementation, analysis, or preparation of any reports or manuscript.

## Results

**Figure 3:**
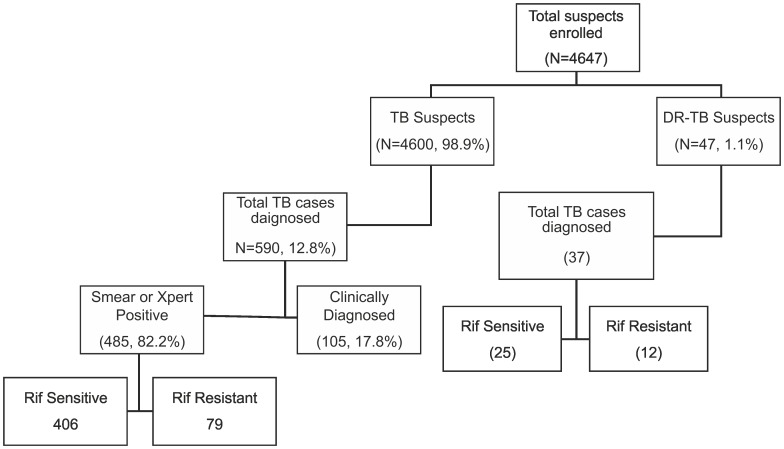
Flow chart of presumptive pediatric TB cases enrolled in the study.

A total of 4,647 pediatric presumptive PTB & DR-TB cases were enrolled across the 18 project sites from March 2012 to December 2013. This included 4,600 (99.0%) presumptive TB cases and 47 (1.0%) presumptive DR-TB cases (Figure 3). The median age of the pediatric presumptive PTB cases (0–14 yrs) was 11 years, with 2,937 (63.8%) in the age group of 10–14 yrs. No significant gender variation was observed among the presumptive TB cases enrolled in the study. A total of 453 (9.8%) presumptive pediatric PTB cases had past history of TB treatment (Table 1).

**Table 1:** TB case detection among presumptive pediatric TB cases (N = 4600).

Variables	Presumptive pediatric TB cases	%	Bacteriologically confirmed PTB (Xpert MTB-Rif/Smear)	%, 95% CI	Clinically diagnosed	%, 95% CI	Total TB Cases (PTB)	%, 95% CI	Rif Resistant TB (among bacteriologically diagnosed)	%, 95% CI
Total	4600	100.0%	485	10.5%, 9.6–11.4	105	2.3%, 1.8–2.7	590	12.8%, 11.8–13.8	79	16.3%, 13.2–19.7
**Gender**										
**Female**	2261	49.2%	336	14.9%, 13.4–16.3	55	2.4%, 1.8–3.1	391	17.3%, 15.7–18.9	58	17.3%, 13.5–21.5
**Male**	2339	50.8%	149	6.4%, 5.4 –7.4	50	2.1%, 1.6–2.7	199	8.5%, 7.4–9.6	21	14.1%, 9.1–20.3
**Age group (years)**										
**Below 5**	304	6.6%	25	8.2%, 5.5–11.7	0	0.0%	25	8.2%, 5.5–11.7	1	4%, 0.2–18.1
**5–9**	1359	29.5%	67	4.9%, 3.8–6.1	35	2.6%, 1.8–3.5	102	7.5%, 6.1–8.9	11	16.4%, 8.9–26.7
**10–14**	2937	63.8%	393	13.4%, 12.1–14.6	70	2.4%, 1.8–2.9	463	15.8%, 14.4–17.1	67	17%, 13.5–21.0
**Past history of anti TB treatment**										
**NA**	8	0.2%	3	37.5%, 10.5–72.2	0	0.0%	3	37.5%, 10.5–72.2	0	0.0%
**No**	4139	90.0%	388	9.4%, 8.5–10.2	99	2.4%, 1.9–2.8	487	11.8%, 10.8–12.7	40	10.3%, 7.5–13.6
**Yes**	453	9.8%	94	20.8%, CI17.2–24.6	6	1.3%, 0.5–2.7	100	22.1%, 18.4–26.07	39	41.5%, 31.8–51.6
**Geographical location**										
**Rural**	1467	32%	65	4.4%, CI3.4–5.5	29	2%, 1.3–2.7	94	6.4%, 5.2–7.7	0	0.0%
**Urban**	2492	54%	347	13.9%, 12.6–15.3	61	2.4%, 1.8–3.11	408	16.4%, 14.9–17.8	71	20.5%, 16.4–24.9
**Tribal & Hilly**	641	14%	73	1.6%, CI9.1–14.3	15	2.3%, 1.3–3.7	88	13.7%, 11.2–16.5	8	11%, 5.2–19.7
**Smear Microscopy**										
**NA**	3	0%	1	33.3%, 1.6–86.8	0	0.0%	1	33.3%, 1.6–86.8	0	0.0%
**Negative**	4375	95%	262	6%, 5.3–6.7	105	2.4%, 1.9–2.8	367	8.4%, 7.5 –9.2	38	14.5%, 10.6–19.1
**Positive**	222	5%	222	100.0%	0	0.0%	222	100.0%	41	18.5%, 15.7–23.9
**Xpert MTB/RIF**										
**NA**	25	1%	5	20.0%, 7.7–38.9	0	0.0%	5	20.0%, 7.7–38.9	0	0.0%
**Indeterminate**	93	2%	0	0.0%	0	0.0%	0	0.0%	0	0.0%
**Negative**	4005	87%	3	0.1%, 0.01–0.2	105	2.6%, 2.1–3.1	108	2.7%, 2.2–3.2	0	0.0%
**Positive**	477	10%	477	100.0%	0	0.0%	477	100.0%	79	16.6%, 13.4–20.1

Abbrevations: Suspects =  number of presumptive PTB patients tested; All PTB =  All diagnosed cases of pulmonary tuberculosis; 95% CI  =  95% confidence interval.

NA = not available,

Overall, 590 (12.8%, CI 11.8–13.8) pediatric PTB cases were diagnosed (Figure 2). Of these, 485 (82.2% CI 78.9–85.1) were bacteriologically-confirmed PTB cases and 105 (17.8% CI 14.8–21.0) were diagnosed based on clinical algorithm alone in the absence of any positive result on Xpert MTB/RIF or smear. The annualized detection rate of bacteriologically-confirmed pediatric PTB cases was 1.5 per 100,000 persons per year (CI 0.6–3.6) in 2011 versus 3.4 per 100,000 persons per year (CI 1.6–7.2) in the programme data during the study period. The rise in the detection rate of bacteriologically-confirmed pediatric PTB cases with Xpert MTB/RIF in the study was found to be statistically significant (p = 0.03) as compared with historical programme data.

Cross-sectional assessment of the bacteriologically-confirmed PTB cases showed that the majority had Xpert MTB/RIF positive results, and negative sputum smear results. Among the 485 bacteriologically-confirmed pediatric PTB cases, 477 (98.4%, CI 96.8–99.2) had a positive TB result on Xpert MTB/RIF and of these 262 (54.9%, CI 50.4–59.3) were Xpert MTB positive and smear negative. An additional 8 cases were diagnosed on smear result alone of which 3 had a negative Xpert MTB/RIF result and 5 could not be tested (Table 1). The overall positivity rate for Xpert MTB/RIF under the study was 10.4% (CI 9.5–11.2), whereas the positivity rate for smear was 4.8% (CI 4.2–5.4). Xpert MTB/RIF had higher positivity rates than smear microscopy in all age groups, averaging a 2.2-fold higher proportion of bacteriologically-confirmed PTB cases (Table 2). This increase in proportion of bacteriologically-confirmed cases was seen to highest in presumptive PTB cases in the age group of 0–4 yrs (Table 2).

**Table 2:** Additional yield on Xpert MTB/RIF as compared to sputum microscopy.

Age group (Years)	Presumptive pediatric TB cases	Smear Positive	%, 95% CI	Xpert MTB/RIF Positive	%, 95% CI	Additional TB cases diagnosed on Xpert MTB/RIF	%, 95% CI
**< 5**	304	4	1.3%, 0.4–3.1	24	7.9%, 6.6–13.2	20	6.6%, 4.1–9.8
**5–9**	1359	19	1.4%, 0.8–2.0	66	4.9%, 3.7–6.0	47	3.5%, 2.5–4.5
**10–14**	2937	199	6.8%, 5.9–7.7	387	13.2%, 11.9–14.4	188	6.4%, 5.5–7.3
**Total**	4600	222	4.8%, 4.2–5.4	477	10.4%, 9.5–11.2	255	5.5%, 4.9–6.2

The proportion of presumptive PTB suspects diagnosed with bacteriologically-confirmed PTB was substantially higher in certain subgroups. Most notably, such cases were more likely to be female (Odd's Ratio (OR) 2.5, CI 2.0–3.1) or have a history of prior anti-TB treatment (OR 2.5, CI 1.9–3.2) (Table 1).

Under the study, a total of 91 rifampicin-resistant pediatric TB cases were diagnosed by offering upfront Xpert MTB/RIF testing. Of these, 79 (86.8%, CI 78.35–92.29) were detected among presumptive pediatric PTB cases and the remaining 12 (13.2% CI 7.34–21.34) were diagnosed from presumptive DR-TB cases (Table 1, Figure 3). Of the 79 rifampicin-resistant cases diagnosed among presumptive PTB cases, 38 were from smear negative. The majority of the rifampicin-resistant cases were detected in urban settings (81, 89.0%, CI 81.2–94.2). The rate of rifampicin resistance among bacteriologically-confirmed PTB cases was seen to be higher in TB cases with previous history of TB treatment (41.5%, CI 31.8–51.6) as compared to patients with no previous history of TB treatment (10.3%, CI 7.5–13.6). Similarly, the rate of rifampicin resistance was higher in bacteriologically-confirmed PTB cases with a history of contact with TB case (26.7%, CI 18.3–36.5) as compared to no history of contact (17.8% CI12.4–24.2).

Out of the 91 rifampicin-resistant cases detected in the study, confirmatory culture DST/LPA results for 48 patients were available at the time of analysis. Of the 48 confirmatory results, 47 were found to be rifampicin resistant giving a PPV of 98% (CI 90.1–99.9). The PPV was similar irrespective of past history of TB treatment and smear positivity (Table 3).

**Table 3:** Positive predictive value compared to either Culture DST and/or LPA in patients with both an Xpert result and Culture DST and/or LPA result.

LPA/Culture DST Rif Res	Rifampicin Resistant	PPV (95% CI)	Rifampicin Sensitive	Total
**Overall PPV**	47	98% (90.1–99.9)	1	48
**Presumptive TB and DR TB cases**				
**New**	21	100% (87–100)	0	21
**Previously treated**	26	96.2% (83–99.8)	1	27
**Smear Microscopy results**				
**Smear Negative**	16	100% (83–100)	0	16
**Smear Positive**	31	97% (85.5–99.8)	1	32

As some of the presumptive pediatric PTB cases enrolled at the study sites were residents of adjacent geographic areas, information on appropriate TB treatment initiation was not available for all diagnosed cases. Of the 406 PTB cases and 91 rifampicin-resistant PTB cases diagnosed, confirmation of treatment initiation was available for 301 (74.1%) and 57 (63%), respectively. At the time of analysis, 115 pediatric PTB cases that were on first line treatment had completed treatment with 93.9% treatment success rate.

## Discussion

In this study, we captured data on fairly large population of pediatric presumptive PTB and DR-TB cases across diverse settings at decentralized levels in India. As observed in similar studies, with the upfront testing on the highly sensitive rapid diagnostic Xpert MTB/RIF more than two-fold the number of bacteriologically-confirmed PTB cases were detected than was seen using smear microscopy alone. This demonstrates the utility of providing upfront access to Xpert MTB/RIF testing to presumptive pediatric PTB cases in line with the recently released guidance from WHO [23]. Similar findings have been documented in studies conducted in South Africa, Uganda, Tanzania, and Vietnam [17]–[20]. Higher diagnostic yield in case detection with use of Xpert MTB/RIF will help in identifying TB patients who are otherwise missed in this age group following current diagnostic practices. Additionally, in the absence of such high sensitivity diagnostic tools the diagnosis of TB in this age group relies heavily on x-ray findings which are not widely available in decentralized locations and requires physically taking the patient to the nearest center with such facility. This represents an additional inconvenience and financial burden on the patients. In contrast, the study design reported here represents a viable alternative wherein a large number of facilities providing care to the pediatric population can be linked to an Xpert MTB/RIF assay by means of specimen transportation.

Factors associated with higher detection rates of PTB under the study were suspects living in urban settings (16.4%), previous history of TB treatment (22.1%) and positive history of contact with a TB case (2.0%). This finding of a higher positivity in patients with history of contact when tested with Xpert MTB/RIF bears resemblance to a similar study conducted in Uganda [19]. Rates of childhood TB are estimated to be the highest among 0–4 years of age [24]. Under the current study the highest gain (two-fold) in PTB case detection rates with Xpert MTB/RIF in comparison to smear microscopy was observed in this (0–4 years) age group. However, low case detection rates have been reported in this age group in previous studies conducted in RNTCP [26]–[27].

While very limited data exists on the expected levels of drug resistance among pediatric TB cases, we record for the first time from India a significant proportion of rifampicin resistant pediatric TB cases (16.3%).The rates of drug resistance among pediatric PTB cases observed in the present study are higher than reported earlier in India [21]–[22]. In our study, the rate of rifampicin resistance was found to be higher in TB cases with previous history of anti-TB treatment, in line with the findings from a cross-sectional study wherein previous history of TB disease and treatment were documented as important risk factor for DR-TB in children [21]-[22]. However, there was wide variation in the detection rates of rifampicin resistance across the sites, with more than 90% of these cases being detected from sites in urban areas, and a single site located in an urban slum area contributing 73% of all diagnosed rifampicin-resistant cases. A recent cross-sectional study of 500 pediatric PTB cases, has reported a rate of 6.8% rifampicin resistance in pediatric PTB cases from this area [21]. The findings of our study highlight that the high levels of rifampicin resistance in pediatric PTB cases are limited to sites in urban areas and not generalized across sites. However, this finding underscores the urgent need for further systematic surveillance of rifampicin resistance levels in the pediatric population, both from case management and epidemiological perspective.

While similar numbers (2,261 females and 2,339 males) of presumptive pediatric cases of both genders were enrolled in the study, the detection rate of PTB were higher in females than in males. However, in similar other studies [19], [21], no gender-specific difference in PTB case detection was observed. Further investigation of possible other co-factors contributing to this finding was beyond the scope of the current study but would be advised for future studies.

A high treatment success rate of 94% was reported in the study among pediatric PTB patients. Similar treatment outcomes have been reported under different studies conducted in various parts of the country [25], [27]–[28].

Based on the findings of the study, a policy decision has been taken by RNTCP to provide access to upfront Xpert MTB/RIF testing to pediatric presumptive TB cases. This strategy is now being rolled out in a phased manner starting with 4 major cities of India [29].

## Conclusion

By offering upfront Xpert MTB/RIF to all the pediatric presumptive TB cases, case detection of pediatric PTB could be doubled compared to the use of smear microscopy. The results from our study clearly demonstrate that Xpert MTB/RIF testing at peripheral settings can be instrumental in meeting the present day challenges of diagnosing TB in pediatric patients. These finding were instrumental in making policy decisions under RNTCP to offer upfront testing on Xpert MTB/RIF for all presumptive pediatric PTB cases.

## Supporting Information

Data S1 **Supporting Data file.** Data S1_Pediatric manuscript data_27072014.
(XLSX)

## References

[1] Newton S, Brent A, Anderson S (2008) Paediatric tuberculosis. Lancet Infectious Disease: Volume 8, (Issue 8): Pages 457–524 (August 2008) Available: http://www.sciencedirect.com/science/article/pii/S1473309908701828. Accessed: 2014 Apr 1.

[2] Kabra S, Lodha R, Seth V (2004) Some current concepts on childhood tuberculosis. Indian J Med Res. 120: 387–397PMID: 15520488, Available: http://www.ncbi.nlm.nih.gov/pubmed/15520488. Accessed: 2014 Apr 1. 15520488

[3] Centre for Disease Control (2014) TB in Children (Global Perspective): 1–4. Available: http://www.cdc.gov/tb/topic/populations/TBinChildren/global.htm. Accessed: 2014 Apr 1.

[4] Jain S, Ordonez A, Kinikar A, Gupte N, Thakar M, et al (2013) Pediatric tuberculosis in young children in India: a prospective study. Biomed Res Int 2013: 783698 10.1155/2013/783698 Available: http://www.pubmedcentral.nih.gov/articlerender.fcgiartid=3872373&tool=pmcentrez&rendertype=abstract. 24386640 PMC3872373

[5] Revised National Tuberculosis Control Program (2013) TB India 2013, RNTCP Annual Status Report Available: http://www.tbcindia.nic.in/pdfs/tb%20india%202013.pdf.

[6] Marais B, Hesseling A, Gie R, Schaaf H, Beyers N (2006) The burden of childhood tuberculosis and the accuracy of community-based surveillance data. International Journal of Tuberculosis and Lung Disease 10: 259–263PMID: 16562704, Available: http://www.ncbi.nlm.nih.gov/pubmed/16562704. 16562704

[7] Kumar A, Gupta D, Nagaraja S, Singh V, Prasad J (2014) Updated National Guidelines for Pediatric Tuberculosis in India, 2012. Indian Pediatr 50: 301–306 Available: http://www.indianpediatrics.net/mar2013/mar-301-306.htm. 10.1007/s13312-013-0085-1 23680604

[8] Avalos G (2012) Classic and New Diagnostic Approaches to Childhood Tuberculosis. Journal of Tropical Medicine Volume 2012 (2012), Article ID 818219, 12 pages. Doi:10.1155/2012/818219, Available: 10.1155/2012/818219. PMC3317187 22529869

[9] Swaminathan S, Rekha B (2010) Pediatric tuberculosis: global overview and challenges. Clin Infect Dis 50 Suppl 3S184–94 10.1086/651490 Available: http://www.ncbi.nlm.nih.gov/pubmed/20397947 Accessed: 2014 March 24. 20397947

[10] Nicol M, Zar H (2011) New Specimens and laboratory diagnostics for childhood pulmonary TB: progress and prospects. *2011 March* 12(1): 16–21 doi:10.1016/j.prrv.2010.09.008 Available: http://www.ncbi.nlm.nih.gov/pmc/articles/PMC3052970/pdf/nihms-238505.pdf. PMC3052970 21172670

[11] Acosta CD, Rusovich V, Harries AD, Ahmedov S, van den Boom M, et al (2014) A new roadmap for childhood tuberculosis. Lancet Glob Heal 2: e15–e17 10.1016/S2214-109X(13)70153-0 Available: http://linkinghub.elsevier.com/retrieve/pii/S2214109X13701530. 25104625

[12] Central TB Division Ministry of Health and Family Welfare, Managing the RNTCP in your area – A training course (Module 1–4). Available: http://tbcindia.nic.in/pdfs/Module%201%20to%204.zip.

[13] Amdekar Y (2010) Consensus statement on childhood tuberculosis Indian Pediatrics, vol. 47, no. 1: pp. 41–55, 2010. Available: http://www.ncbi.nlm.nih.gov/pubmed/20139477 10.1007/s13312-010-0008-3 20139477

[14] Central TB Division Ministry of Health and Family Welfare,New Delhi, India, Revised National TB Control Programme (2012) Guidelines on Programmatic Management of Drug Resitant TB (PMDT) in India. Available: http://tbcindia.nic.in/pdfs/Guidelines%20for%20PMDT%20in%20India%20-%20May%202012.pdf

[15] World Health Organization. Geneva (2013) Definitions and reporting framework for tuberculosis -2013 revision. S.n.p.3. Available: http://apps.who.int/iris/bitstream/10665/79199/1/9789241505345_eng.pdf.

[16] Central TB Division Ministry of Health and Family Welfare, National guidelines on diagnosis and treatment of Pediatric Tuberculosis (2012), Available: http://tbcindia.nic.in/Paediatric%20guidelines_New.pdf.

[17] Nicol MP, Workman L, Isaacs W, Munro J, Black F, et al (2011) Accuracy of the Xpert MTB/RIF test for the diagnosis of pulmonary tuberculosis in children admitted to hospital in Cape Town, South Africa: a descriptive study. 3099: 1–6 10.1016/S1473-3099(11)70167-0 Available: http://www.ncbi.nlm.nih.gov/pubmed/21764384. PMC4202386 21764384

[18] Sekadde M, Wobudeya E, Joloba M L, Sengooba W, Harriet K, at el (2013) Evaluation of the Xpert MTB/RIF Test for the Diagnosis of Childhood Pulmonary Tuberculosis in Uganda: a cross sectional diagnostic study, BMC Infectious Diseases 2013. 13: 133 Available: http://www.biomedcentral.com/content/pdf/1471-2334-13-133.pdf. 10.1186/1471-2334-13-133 PMC3602671 23497044

[19] Rachow A, Clowes P, Saathoff E, Mtafya B, Michael E, et al (2012) Increased and expedited case detection by Xpert MTB/RIF assay in childhood tuberculosis: a prospective cohort study. Clin Infect Dis 54: 1388–1396 10.1093/cid/cis190.Epub2012Apr3 Available: http://www.ncbi.nlm.nih.gov/pubmed/22474220 Accessed: 2014 Apr 1. 22474220

[20] Nhu NT, Ha DT, Anh ND, Thu do DA, Duong TN, et al (2011) Evaluation of Xpert MTB/RIF and MODS assay for the diagnosis of pediatric tuberculosis. Lancet Infect Dis 2011 Nov; 11(11): 819–24 10.1016/S1473-3099(11)70167-0.Epub2011Jul19 Available: http://www.pubmedcentral.nih.gov/articlerender.fcgiartid=3562258&tool=pmcentrez&rendertype=abstract Accessed 1 April 2014. Accessed: 2014 Apr 1.

[21] Shah I, Chilkar S (2012) Clinical profile of drug resistant tuberculosis in children. Indian Pediatric 2012 Sep; 49(9): 741–4. Epub 2012 Jun 10, Available: http://www.ncbi.nlm.nih.gov/pubmed/22728624. 10.1007/s13312-012-0158-6 22728624

[22] Shah I (2012) DRUG RESISTANT TUBERCULOSIS IN CHILDREN IN INDIA. Pediatric Oncall [serial online] 2012[cited 2012 January 1]; 9. Art #27, DOI:10.7199/ped.oncall.2012.27, Available: http://www.pediatriconcall.com/Journal/Article/FullText.aspxartid=490&type=J&tid=&imgid=&reportid=365&tbltype=

[23] World Health Organisation-Policy statement (2011) Automated Real-time Nucleic Acid Amplification Technology for Rapid and Simultaneous Detection of Tuberculosis and Rifampicin Resistance: Xpert MTB/RIF System Available: http://whqlibdoc.who.int/publications/2011/9789241501545_eng.pdf. 26158191

[24] Central TB Division Ministry of Health and Family Welfare-Revised National Tuberculosis program (2012) National Strategic Plan for Tuberculosis Control 2012–2017. pp. 100-105,

[25] Satyanarayana S, Shivashankar R, Vashist R, Chauhan L, Chadha S, et al (2010) Characteristics and programme-defined treatment outcomes among childhood tuberculosis (TB) patients under the national TB programme in Delhi. PLoS One 5: e13338 10.1371/journal.pone.0013338 Available: http://www.pubmedcentral.nih.gov/articlerender.fcgiartid=2953513&tool=pmcentrez&rendertype=abstract Accessed: 2014 Apr 1. 20967279 PMC2953513

[26] Thakur R (2014) Characteristics of childhood tuberculosis patients registered under RNTCP in Varanasi, Uttar Pradesh- Indian J Public Health: 2–5. 57: 36–9 DOI: 10.4103/0019-557X.111367 , Available: 10.4103/0019-557X.111367 http://www.ijph.in/article.aspissn=0019557X;year=2013;volume=57;issue=1;spage=36;epage=39;aulast=Ruchi, Available: http://www.ijph.in/article.aspissn=0019557X;year=2013;volume=57;issue=1;spage=36;epage=39;aulast=Ruchi , Accessed: 2014 Jan 22 23649142

[27] Arora V, Agarwal S (2005) Chapter 12-Paediatric Tuberculosis: An Experience from LRS Institute of Tuberculosis and Respiratory Diseases. Tuberculosis Control In India.Vol. 330: pp. 115–118. Available: http://tbcindia.nic.in/pdfs/Tuberculosis%20Control%20in%20India12.pdf.

[28] Nelliyanil M, Sharada MP, Joseph N, Basagoudar SS, Jayaram S, et al (2014) A study of the socio-demographic profile and treatment outcome of paediatric tuberculosis patients in Bangalore Mahanagar Palike area. Indian J Tuberc 2012 Oct; 59(4): 207–13 Available: http://www.ncbi.nlm.nih.gov/pubmed/23342540. 23342540

[29] http://www.finddiagnostics.org/media/press/140430.html, Accessed: 2014 May 3.

